# Variable E-field properties of dual-site tACS with phase lags

**DOI:** 10.1162/IMAG.a.1068

**Published:** 2026-01-05

**Authors:** Silvana Huertas-Penen, Maria Carla Piastra, Oula Puonti, Bettina C. Schwab

**Affiliations:** Biomedical Signal and Systems, Electrical Engineering, Mathematics and Computer Science, University of Twente, Enschede, the Netherlands; Clinical Neurophysiology, Technical Medical Centre, University of Twente, Enschede, the Netherlands; Athinoula A. Martinos Center for Biomedical Imaging, Massachusets General Hospital and Harvard Medical School, Boston, MA, United States; Danish Research Centre for Magnetic Resonance, Copenhagen University Hospital - Amager and Hvidovre, Copenhagen, Denmark; Department of Neurology, University Medical Center Hamburg Eppendorf, Hamburg, Germany

**Keywords:** dual-site transcranial alternating current stimulation (ds-tACS), electric field, functional connectivity, phase lag, primary motor cortex, high-definition montages

## Abstract

Dual-site transcranial alternating current stimulation (ds-tACS) enables the modulation of interregional functional connectivity by introducing a phase lag between the stimulating currents. However, overlapping electric fields (E-fields), particularly in closely spaced cortical targets like the primary motor cortices (M1s), may unintentionally alter E-field characteristics and confound the interpretation of functional connectivity modulation. We aimed to systematically evaluate how different phase lags affect key E-field characteristics when using high-definition ds-tACS, particularly when targeting the M1s. We sought to determine which montage configuration best preserved stable E-field characteristics and investigated whether individualised montage selection could enhance control over E-field consistency. We used individualised finite-element method simulations based on MRI-derived head models to quantify the effects of different phase lags on E-field characteristics. E-field magnitude, normal component, spatial distribution, direction, and effective stimulation area were assessed for nine montages and eight phase lags. All E-field properties, including E-field peak magnitude, peak magnitude of the normal component, redistribution, difference to optimal direction, and effective area of stimulation, were modulated significantly across different phase lags for all tested montages. Furthermore, we found substantial inter-individual variability in all E-field properties. Individual selection of montages improved critical properties, particularly the E-field direction. In contrast to common assumptions, variations in the phase lag can significantly affect key E-field properties of high-definition ds-tACS. Therefore, we recommend considering modulations of the E-field characteristics when comparing physiological or behavioural effects of ds-tACS at different phase lags. Moreover, given the high inter-individual variability, we suggest the individualisation of montages to the most relevant E-field property.

## 1 Introduction

Transcranial alternating current stimulation (tACS) noninvasively delivers alternating electric currents to the scalp, generating weak electric fields (E-fields) in cortical regions. These weak E-fields have recently been shown to phase-specifically modulate neural activity (Fiene et al., 2020; Johnson et al., 2020; M. R. Krause et al., 2019). A specific variant, dual-site (ds) tACS, introduces a phase lag between the stimulation currents of the two targeted brain regions, changing the relative timing of stimulation between the two regions. This relative timing translates into different directions of the E-fields across phase lag conditions.

Building on this principle, ds-tACS can steer the timing of the E-field across two target regions, inducing phase-specific polarisation of pyramidal neurones (Saturnino et al., 2017). This polarisation can change the timing of spikes, causing neurones to fire in alignment with the phase of the applied E-field (Fiene et al., 2020; Johnson et al., 2020; M. R. Krause et al., 2019). By aligning spikes to the E-field phases in the targeted regions, ds-tACS can synchronise oscillatory activity with arbitrary phase lags between regions, thereby modulating interregional functional connectivity (FC) (Gann et al., 2025; Helfrich et al., 2014; Lafleur et al., 2020; Preisig & Hervais-Adelman, 2022; Saturnino et al., 2017; Schwab et al., 2019; Seo et al., 2024; Tran et al., 2021; Zhang et al., 2023).

Such modulation is of great interest for both basic and clinical neuroscience, as it allows the investigation of the causal influences of FC on behaviour and may pave the way for readjusting pathological levels of FC in diseases. Ds-tACS has thus been widely applied, for example, for the modulation of FC between frontal and temporal regions (Reinhart & Nguyen, 2019) or motor cortical networks (Heise et al., 2019). In sum, a number of studies have demonstrated that ds-tACS can lead to changes in FC and behaviour (Cabral-Calderin et al., 2016; Reinhart & Nguyen, 2019; Weinrich et al., 2017), even demonstrating robust correlations between the two (Fujiyama et al., 2023; Lebihan et al., 2025; Meng et al., 2023; Tan et al., 2025; Violante et al., 2017; Zhu et al., 2025).

Nevertheless, ds-tACS is often applied with the assumption of spatially separated E-fields. Separated E-fields imply that phase lag manipulation would influence only the timing and, therefore, the relative direction of the two E-fields, while leaving the E-field magnitude, spatial properties, and stimulated area unchanged. However, ds-tACS typically leads to at least partially overlapping E-fields. These overlaps can result in complex spatial interactions, where phase lag–dependent interactions unintentionally alter E-field characteristics (Saturnino et al., 2017). Consequently, these changes in E-field properties may modulate not only FC but also other neural properties, such as oscillatory power, challenging the goal of selectively targeting FC.

To improve the spatial precision of stimulation and reduce unintended effects, high-definition (HD) montages have been developed (Gbadeyan et al., 2016). Compared with conventional two-electrode setups, HD montages provide more focal stimulation (Datta et al., 2009; Heise et al., 2016; Morales-Quezada et al., 2019). While HD montages represent a significant advance, they do not fully eliminate the issue of overlapping E-fields (Heise et al., 2016; Saturnino et al., 2017). This overlap is particularly relevant when stimulating close-by brain regions such as the primary motor cortices (M1s). Although previous modelling work has described the problems caused by overlapping E-fields (Saturnino et al., 2017), the quantitative extent to which phase lags affect E-field characteristics remains poorly understood. Furthermore, the E-field properties of phase lags beyond zero and π have not yet been investigated.

Thus, in this study, we aimed to systematically quantify the effects of different phase lags between stimulation currents at two sites on key E-field characteristics of HD ds-tACS. Here, we use the term ds-tACS specifically to refer to HD ds-tACS with applied phase lags. We focused on the M1s, which are common targets for neuromodulation (Giustiniani et al., 2019; Heise et al., 2019; V. Krause et al., 2016; Lafleur et al., 2020; Liu et al., 2025; Miyaguchi et al., 2018, 2019; Nakazono et al., 2016; Rjosk et al., 2016; Rumpf et al., 2019; Wach et al., 2013). We constructed individualised finite-element method (FEM) simulations based on MRI-derived head models using open-access data from the Human Connectome Project (HCP) (Van Essen et al., 2013). We then assessed the phase lag-dependent variations in the E-field properties, including field magnitudes, spatial distribution, direction, and effective stimulation area for nine montages and eight phase lags. Additionally, we sought to determine which HD montage configuration best preserved stable E-field characteristics, ensuring consistency in the stimulation effects. Finally, we investigated whether individualised montage selection could enhance control over E-field consistency and thereby improve the interpretability of ds-tACS interventions.

## 2 Materials and Methods

### 2.1 Dataset

We used n =18 datasets from the HCP (Van Essen et al., 2013), which included preprocessed T1 and T2 MRI scans acquired at 3T (Van Essen et al., 2013). The 18 individuals (nine females) were randomly selected, with six from each age group: 22–25, 26–30, and 31–35 years. All data used were open access. The IDs of the selected individuals are listed in Supplemental Material 1, Table S1.

### 2.2 Electrode montages

**Fig. 1.**
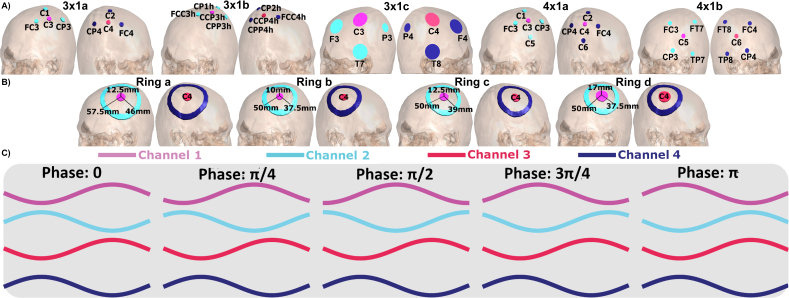
(A) MSE montage configurations. (B) Ring montage configurations. (C) Injected currents for different phase lags.

We simulated nine typical motor cortex montages: five HD multiple small electrodes (MSE) montages (Fig. 1A) and four HD ring electrode montages (Fig. 1B). The ring montages varied in electrode size (Kaiser et al., 2025; Preisig & Hervais-Adelman, 2022; Salamanca-Giron et al., 2021; Saturnino et al., 2017), with the central electrodes positioned above the M1s (C4 in the right hemisphere and C3 in the left). Each ring montage included a ring electrode with varying inner and outer radii around the central electrode, as detailed in Appendix A, Table A7. The electrodes were selected from commercially available sizes.

Two common MSE electrode configurations were used: a central electrode surrounded by three electrodes (3 × 1) (Gann et al., 2025; Reinhart & Nguyen, 2019; Saturnino et al., 2021; Seo et al., 2024) or four electrodes (4 × 1) (Asamoah et al., 2019; Dmochowski et al., 2011; Gebodh et al., 2021; Hu et al., 2022; Meng et al., 2023; Saturnino et al., 2017; Schwab et al., 2019; Soleimani et al., 2022; Spooner & Wilson, 2023; Yang et al., 2023). The positions of the electrodes were based on the extended SPM12 10-20 EEG system, available in SimNIBS 4.0 (Puonti et al., 2020; Thielscher et al., 2015). Table A8 in Appendix A lists the electrode positions for each montage. All electrodes had a diameter of 12 mm and a height of 1 mm (StarStim, Neuroelectrics, Spain), except for those used in the 3 × 1c montage, which followed the specifications described by Gann et al. (2025).

The central electrodes in both montage types delivered currents in the polarity opposite to those of the surrounding electrodes. In the MSE montages, the current of the central electrode was three or four times higher than that of the surrounding electrodes, whereas the currents were equal in the ring montages. This current distribution ensured a net current of zero. Table A9 in Appendix A lists the conductivity values for each compartment (Puonti et al., 2020).

### 2.3 Simulation framework

The charm function of SimNIBS version 4.0 (Puonti et al., 2020) was used to segment the MRI data into nine compartments and to construct meshes. We then simulated E-fields in SimNIBS (Thielscher et al., 2015). Based on Saturnino et al. (2017), E-fields of ds-tACS were calculated using three tDCS simulations per dataset and montage based on linear superposition:

$$\begin{array}{cccc} \begin{array}{l} E\left(p,t\right)=I_{o}(E_{R1}\left(p\right)sin\left(2\pi ft\right)-E_{C1}\left(p\right)sin\left(2\pi ft\right)\\ \;\;\;\;\;\;\;\;\;\;\;\;\;+E_{R2}\left(p\right)sin\left(2\pi ft+\phi \right)) \end{array} & & & \end{array}$$ (1)

where $I_{o}$ is the amplitude of the applied current, p is the spatial position, t is the time, and f is the stimulation frequency. $E_{R1}\left(p\right)$ $E_{C1}\left(p\right)$, and $E_{R2}\left(p\right)$ denote the E-fields obtained from three separate tDCS simulations, each using a common return electrode (C2), located in the left hemisphere.

**Fig. 2.**
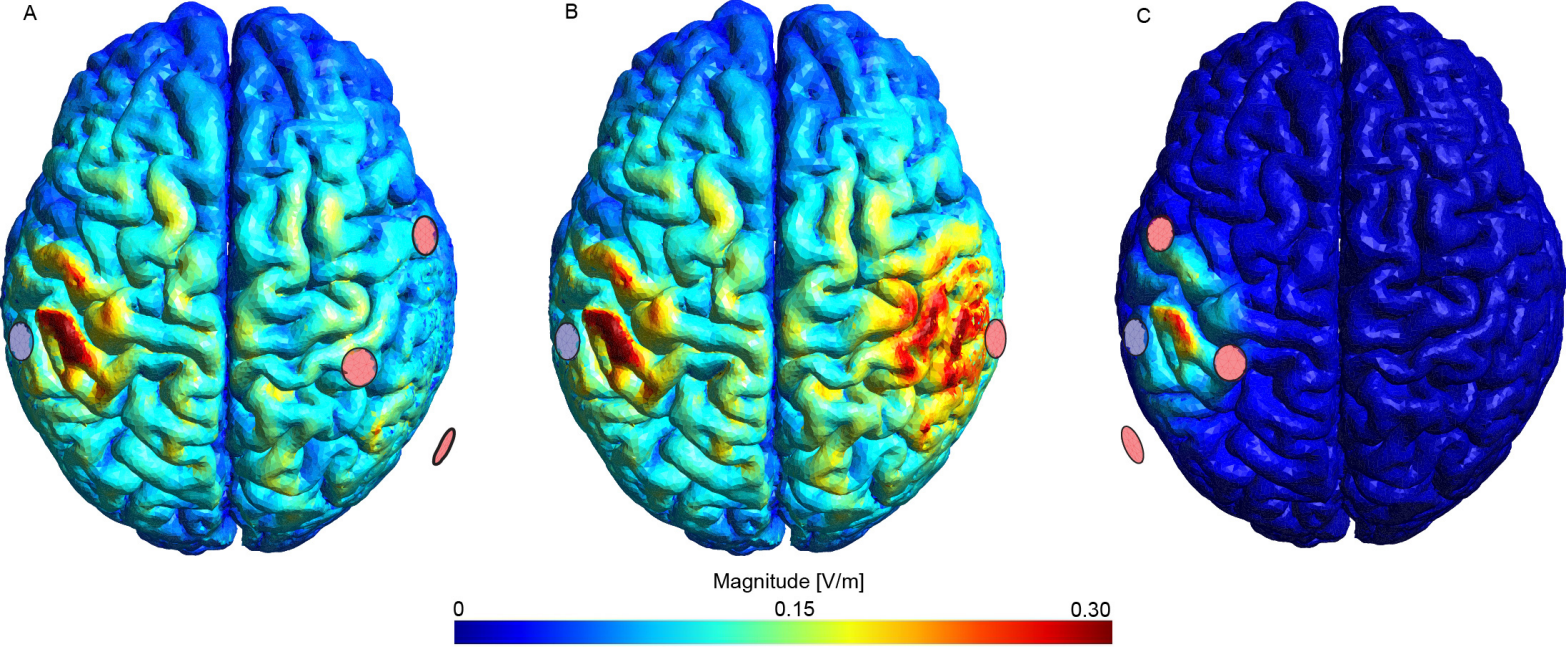
E-field magnitudes for the tDCS simulations with the central left electrode as the return common electrode, for an example individual (970764). (A) $E_{R1}$ : Surrounding the electrodes in the right hemisphere. (B) $E_{C1}$ : Central electrode in the right hemisphere. (C) $E_{R2}$ : Surrounding electrodes in the left hemisphere. The red electrodes indicate the active sources (R1, C1, or R2), and the blue electrode represents the shared return electrode (C2). The E-fields of tACS were computed as linear superpositions of the depicted E-fields.

- $E_{R1}\left(p\right)$ corresponds to the E-field generated by currents between the return electrode (C2) and the surrounding right electrodes (R1) as shown in Figure 2A.
- $E_{C1}\left(p\right)$ represents the E-field produced by current between the return electrode (C2) and the central right electrode (C1) as shown in Figure 2B.
- $E_{R2}\left(p\right)$ corresponds to the E-field generated by currents between the return electrode and the surrounding left electrodes (R2) as shown in Figure 2C.

Phase lags were introduced by adjusting the phase of $E_{R2}$ , denoted as ϕ.

**Fig. 3.**
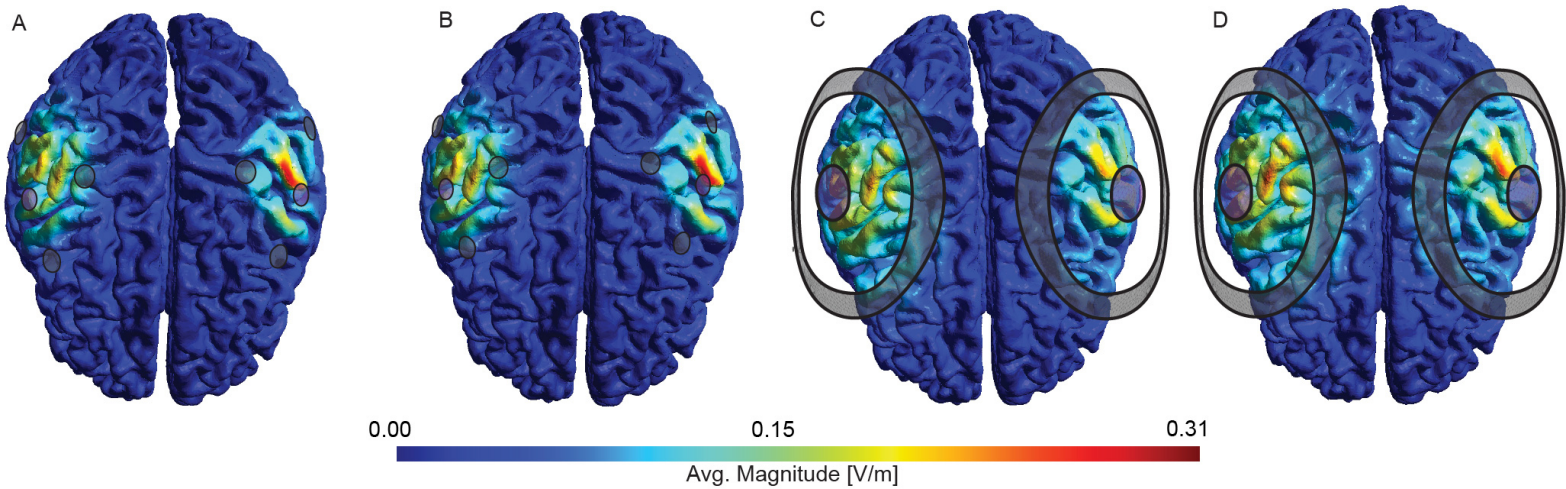
Time-average E-Field magnitudes for an example individual (970764). (A) 3 × 1a MSE montage at phase lag zero (B) and at phase lag $\frac{3\pi }{4}$. (C) Ring b montage with a phase lag of zero; (D) and a phase lag $\frac{3\pi }{4}$. The colour of the electrodes reflects current polarity at one time step.

The E-fields were computed for 24 equally spaced time steps. This procedure is required when including phase lags other than zero and π, as in the remaining cases, there is not one time step where the maximum current is reached simultaneously in all channels. Thus, E-fields for all phase lags were simulated across all time steps (see Section 2.4). The simulations were conducted with a maximum peak-to-peak current of 4 mA and a frequency of 20 Hz. Because the E-fields scale linearly with the amplitude, our results can be directly translated into experimental settings with different current intensities. We explored eight phase lags ranging from 0 to 7/4 π, thus equally sampling the entire cycle. Figure 1C illustrates sinusoidal stimulation for the first five phase lags; the remaining phase lags are symmetric around π. Figure 3A-D show the average E-field magnitude over time for one individual and two exemplary phase lags (0 and $\frac{3\pi }{4}$) for one MSE montage and one ring montage.

### 2.4 E-field metrics relevant to FC modulation

To quantify the E-field characteristics, we applied thresholds to minimise the influence of numerical noise and simulation inaccuracies (Saturnino et al., 2017; Van Hoornweder et al., 2023). Values exceeding the 99.9th percentile of either the E-field magnitude or the absolute value of the component normal to the cortical surface (normal component) were eliminated to reduce the influence of numerical inaccuracies that can occur in the FEM simulations. Conversely, values below 2% of the absolute 99.9th percentile of the E-field were set to zero to suppress negligible field contributions, where the simulated direction and distribution may be unreliable. These very weak E-fields are also below the levels expected to lead to physiological effects (Alekseichuk et al., 2022). Together, these thresholds ensure that subsequent analyses focus on the meaningful range of E-field values while avoiding numerical inaccuracies or numerically unstable values. The resulting data were used to compute the relevant metrics for each montage and phase lag.

**Fig. 4.**
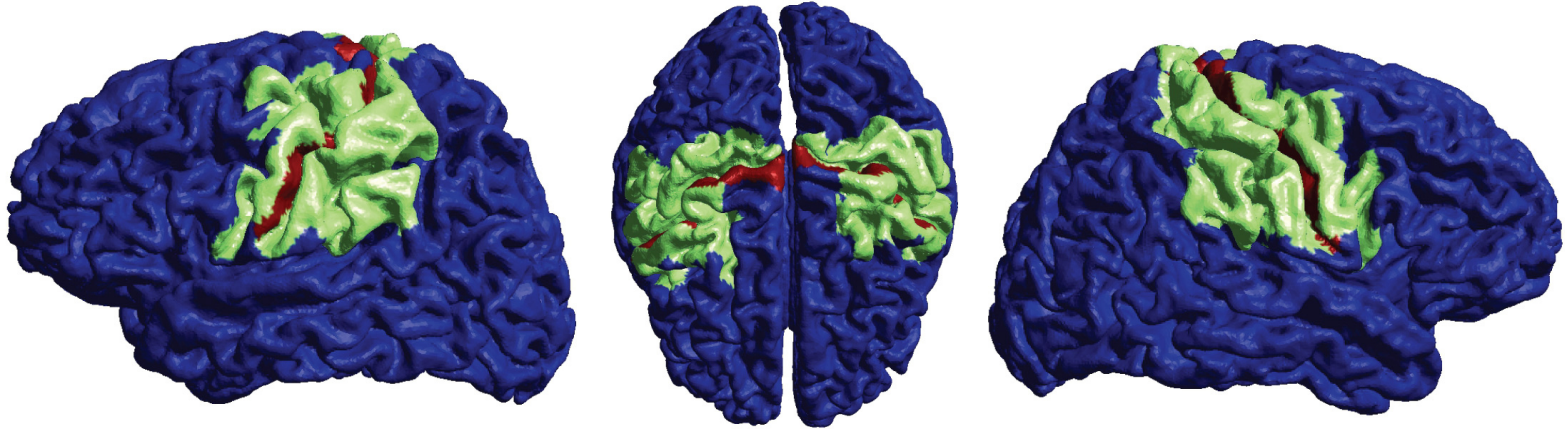
Surrounding grey matter sheet with minimum average E-field magnitudes of 0.01 V/m (green), grey matter sheet of the M1s (red).

For each individual, metrics were calculated independently for the grey matter sheet of the M1s and the surrounding grey matter sheet (composed of 28 additional regions). The selected surrounding regions received an average E-field magnitude of 0.01 V/m or greater in all montages, both hemispheres, and all individuals (Fig. 4). Other regions were neglected in the analysis to ensure an overall physiologically meaningful field magnitude and to mitigate the impact of potential inaccuracies in the simulations. The regions were based on the Glasser Atlas (Glasser et al., 2016). A full list of the selected regions is provided in Appendix B, Table A10.

We investigated the following metrics, each calculated twice: once for the M1s, and once for the surrounding regions, except the effective area of stimulation that was only calculated for each M1 independently:

#### 2.4.1 Magnitudes

a) Peak magnitude of E-field: A commonly used measure for estimating the E-field strength (Saturnino et al., 2017).

b) Peak magnitude of E-field normal component: A commonly used measure for estimating the “effective” peak magnitude of the E-field, that is, in the direction of cortical dendrites, and thus normal to the grey matter sheet (Saturnino et al., 2017).

Both types of peak magnitudes were calculated at each time step, and then the maximum peak across time steps were selected. Focusing on cortex-normal components captures the orientation most relevant for inducing polarisation of pyramidal neurones along their principal dendritic axes. Although the overall magnitude of the E-field contributes to activation in TMS (Weise et al., 2023), the normal component is especially important for tACS: modelling and empirical studies indicate this across multiple lines of evidence. Multi-scale simulations show that the somatic polarisation of pyramidal neurones during tACS is largely determined by the normal component (Huang et al., 2025), cortical response analyses reveal a strong correlation with somatic polarisation (Chung et al., 2022), and empirical evidence suggests that the normal component is more predictive of neurophysiological effects than field magnitude alone (Antonenko et al., 2019).

#### 2.4.2 Redistribution

The relative distance measure (RDM) quantifies the dissimilarity between the spatial distribution of the E-field at a phase lag of zero and at a nonzero phase lag (Meijs et al., 1989; Saturnino et al., 2017). This dissimilarity can be formalised as, for each time-step as:

$$\text{RDM}\left(t\right)=\left\|\;\frac{\left|E_{n}^{0}\left(t\right)\right|}{∥E_{n}^{0}\left(t\right)∥}-\frac{\left|E_{n}^{\phi }\left(t\right)\right|}{∥E_{n}^{\phi }\left(t\right)∥}\;\right\|$$ (2)

where t indicates the time step. $E_{n}^{0}$ and $E_{n}^{\phi }$ are vectors of the E-fields oriented normally to the centre of the grey matter sheet for a phase lag of zero (0) and for other phase lags (ϕ), respectively. The single vertical bars |⋅| denote the element-wise absolute values of the normal components, and the ∥⋅∥ indicate the Euclidean norm.

After computing RDM(t) for each time step, we averaged these values to obtain the overall RDM. The maximum value of the RDM is $\sqrt{2}$, indicating that the normal component distributions of the two-phase lags do not overlap. An RDM value of zero signifies an identical distribution of the normal component of the E-field, which is the desired situation for ds-tACS.

#### 2.4.3 Direction

a) The Dot Product (DotP) quantifies changes in the direction of the normal component of the E-field between the zero phase-lag condition and other conditions (Saturnino et al., 2017). For each time step t, the DotP was computed using vectors of normal E-field components across space as:

$$\text{DotP}\left(t\right)=\frac{E_{n}^{0}\left(t\right)\;\cdot \;E_{n}^{\phi }\left(t\right)}{∥E_{n}^{0}\left(t\right)∥\;∥E_{n}^{\phi }\left(t\right)∥}$$ (3)

The overall DotP was then obtained by averaging DotP(t) across all time steps t. If the E-fields have the opposite direction in all the analysed nodes, DotP(t) is -1. By contrast, if the E-field has the same direction in all the analysed nodes, DotP(t) becomes 1.

b) We define the difference between the ideal DotP ($IDotP_{0,\phi }$ ) and the obtained one ($ODotP_{0,\phi }$ ) between the E-fields of phase lags zero and ϕ as

$$\Delta DotP=\left|IDotP_{0,\phi }-ODotP_{0,\phi }\right|$$ (4)

The ideal values were calculated as described in Supplemental Material 1, Section B.

#### 2.4.4 Effective area of stimulation

We quantified the focality of the normal component of the E-field within the grey matter sheet of each M1 region using the effective area of stimulation (Saturnino et al., 2019):

$$|E{|}_{area}\left(t\right)=\frac{\sum_{i=1}^{m}\;|E_{norm,i\left(t\right)}|g_{i\;}\text{*}\sum_{j=1}^{n}\;G_{j}}{\sum_{j=1}^{n}\;E_{norm,j\left(t\right)}*\;G_{j}}$$ (5)

For each time step t, the effective area of stimulation $|E{|}_{area}\left(t\right)$ was computed. The overall effective area of stimulation was obtained by averaging $|E{|}_{area}\left(t\right)$ across all time steps t.

In equation 5, $E_{norm,i}$ represents the vector of the normal components of the E-field that are equal to or greater than 50% of the peak normal component, and $g_{i}$ corresponds to the area of the nodes of these E-fields. $G_{j}$ denotes the area of all nodes in the region of interest, whereas $E_{norm,j}$ represents the vector of the normal components of all E-fields within the region of interest. Thus, the effective area of stimulation $|E{|}_{area}$ is the area in the target region where the normal components of the E-field are equal to or greater than 50% of the maximum E-field normal components on the surrounding grey matter sheet, including the M1s.

### 2.5 Variability of metrics

The coefficient of variation (CV) was used to quantify the inter-individual variability for each metric:

$$CV=\frac{SD}{Average}*100\%$$ (6)

The standard deviation (SD) and average values across individuals were calculated for each metric and each montage and then averaged across all phase lags.

Additionally, we tested the potential of individualising montages based on a preselected set of montages. The best-performing montage was selected for each individual. Both the metric of interest and its CV were then calculated across individuals using these selected montages and averaged over all phase lags. Individualisation tests were performed only for RDM and ΔDotP, as these metrics capture E-field direction and redistribution, which are both considered essential to the specificity of FC modulation by ds-tACS. In contrast, optimisation of peak magnitude and focality, while relevant for tACS in general, has been extensively addressed in prior studies and is already implemented in SimNIBS (Dmochowski et al., 2011; Saturnino et al., 2015, 2019; Weise et al., 2025).

### 2.6 Spherical head model

In addition to the individualised head models, we also simulated a spherical model provided in SimNIBS 4.0 (Puonti et al., 2020) (a detailed description of the model can be found in Supplemental Material 1, Section C). This simplified model created a controlled environment to investigate optimal E-field characteristics with minimal overlap of E-fields and to verify the consistency of our simulation pipeline.

### 2.7 Statistical testing

For the peak E-field estimates, we selected the maximum value observed across all time steps. For the other metrics, we calculated the average value across time steps.

To assess whether the metrics exhibited significant modulation across the phase lags, we used the weighted Hermans-Rasson (WHR) test (Landler et al., 2019). For each metric, montage, and region (M1s or surrounding regions), we generated a null distribution by randomly permuting the phase lags 10,000 times and recomputing the WHR statistic for each permutation. The empirical WHR statistic from the unshuffled data was then compared to this null distribution to obtain a p-value. Bonferroni–Holm correction was applied to control for multiple montage comparisons. A metric was considered significantly modulated by the phase lags when the corrected p-value was < 0.05.

## 3 Results

### 3.1 Ideal values in a sphere model

We first investigated all metrics under ideal conditions with small ring electrode montages on a spherical model (Supplemental Material 1, Fig. S2A). As expected, the peak magnitude of the E-field normal components were low but stable over the phase lags. The RDM was almost zero for all the phase lags. Furthermore, the DotP was very close to the ideal value. In summary, under ideal conditions, it is possible to separate the two E-fields. Further details of the results are provided in Supplemental Material 1, Figure S2B-D.

### 3.2 E-field magnitudes slightly depend on the phase lag

**Fig. 5.**
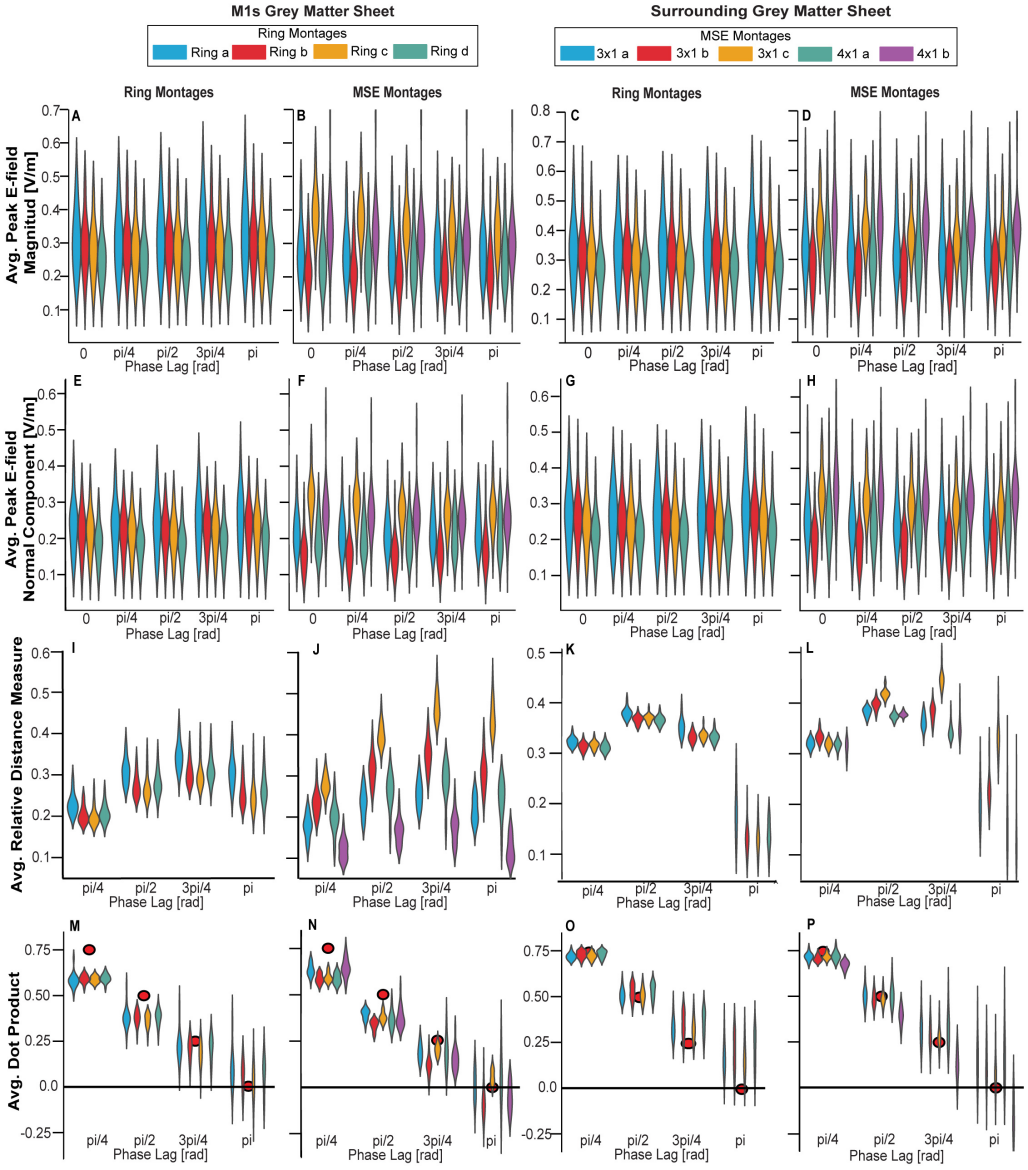
Summary of E-field metrics. Columns 1 and 3 show results for the ring montages, while columns 2 and 4 correspond the MSE montages; columns 1–2 correspond to the M1s grey matter sheet; and columns 3–4 to the surrounding grey matter sheet. (A–D) Maximum peak E-field magnitude, (E–H) maximum peak E-field normal component, (I–L) average RDM, (M–P) average DotP. Red dots indicate the ideal DotP. Details can be found in Supplemental Material 1, Section D.

Next, we studied the metrics for realistic head models. As the first key question, we examined how the E-field magnitude varied across the phase lags for different montages. Figure 5 shows the peak E-field magnitude (panels A-D) and peak inward normal component (panels E-H) for all montages (for details on each participant’s measures, see Supplemental Material 1, Figs. S3-S4). Tables 1 and 2 present the average values per montage. Notably, different average properties, such as the largest mean and the smallest differences across phase lags, are optimal for different montages. In other words, no single montage maintained optimal E-field properties across all phase lags. The peak E-field magnitude and peak magnitude inward normal component, computed across all individuals and montages for both the M1s and the neighbouring regions, were slightly but significantly influenced by the phase lag (all corrected p-values $<10^{-3}$ , WHR >4.04 for the M1s and WHR >0.51 for the surrounding regions).

**Table 1.** Overview of population-average peak E-field magnitudes per montage.

	3** × **1a	3** × **1b	3** × **1c	4** × **1a	4** × **1b	Ring a	Ring b	Ring c	Ring d
Avg. peak magnitude surrounding regions [V/m]	0.347	0.265	0.395	0.333	**0.419**	0.344	0.327	0.310	0.275
Diff max. - min. peak magnitude surrounding regions [V/m]	0.440	0.369	0.351	0.471	0.506	0.411	0.429	0.375	**0.321**
Avg. peak magnitude M1s [V/m]	0.278	0.221	**0.360**	0.252	0.332	0.319	0.298	0.284	0.257
Diff max. - min. peak magnitude M1s [V/m]	0.336	0.309	0.376	0.321	0.539	0.407	0.360	0.331	**0.284**

The average/difference between the maximum and minimum across all phase lags and individuals are shown. The best montage for every measure (largest mean and smallest difference) is shown in bold.

**Table 2.** Overview of population-average peak E-field normal components per montage.

	3 × 1a	3 × 1b	3in1c	4 × 1a	4 × 1b	Ring a	Ring b	Ring c	Ring d
Avg. peak normal surrounding regions [V/m]	0.260	0.192	0.310	0.252	**0.328**	0.270	0.255	0.243	0.215
Diff max. - min. peak normal surrounding regions [V/m]	0.346	0.280	0.289	0.362	0.422	0.329	0.341	0.300	**0.263**
Avg. peak normal M1s [V/m]	0.220	0.162	**0.287**	0.205	0.270	0.241	0.220	0.214	0.187
Diff max. - min. peak normal M1s [V/m]	0.243	0.256	0.292	0.226	0.404	0.312	0.257	0.252	**0.214**

The average/difference between the maximum and minimum across all phase lags and individuals are shown. The best montage for every measure (largest mean and smallest difference) is shown in bold.

### 3.3 E-field redistributions considerably change with the phase lag

The second key question focuses on investigating the extent to which the E-field distribution changes across phase lags. Figure 5 (panels I-L), presents the RDM for the MSE and ring montages, comparing the dissimilarity in the E-field distribution between the zero-phase lag and the other phase lags. These comparisons were conducted across the surrounding grey matter sheet (Fig. 5, panels K-L) and grey matter sheet of the M1s (Fig. 5, panels I-J), with average properties described in Table 3 (for details on each participant’s measures, see Supplemental Material 1, Fig. S5). We observed a redistribution of E-fields from phase lag zero to all other phase lags, with the extent of this redistribution being strongly dependent on the phase lag for both the surrounding grey matter sheet and M1s (all corrected p-values $<10^{-3}$ , WHR >2.69 for the surrounding regions and WHR >4.75 for the M1s).

**Table 3.** Overview of population-average RDM values per montage.

	3 × 1a	3 × 1b	3 × 1c	4 × 1a	4 × 1b	Ring a	Ring b	Ring c	Ring d
Avg. RDM surrounding regions	0.333	0.351	0.386	0.319	0.317	0.327	**0.307**	0.310	0.308
Max. RDM surrounding regions	0.396	0.416	0.497	0.388	0.413	0.408	**0.383**	0.389	0.386
Avg. RDM M1s	0.222	0.302	0.391	0.247	**0.148**	0.296	0.260	0.256	0.266
Max. RDM M1s	0.315	0.425	0.554	0.341	**0.169**	0.421	0.373	0.390	0.389

The average/maximum across all phase lags are shown. The best montage for every measure (smallest value) is shown in bold.

### 3.4 The effective area of stimulation depends on the phase lag

**Fig. 6.**
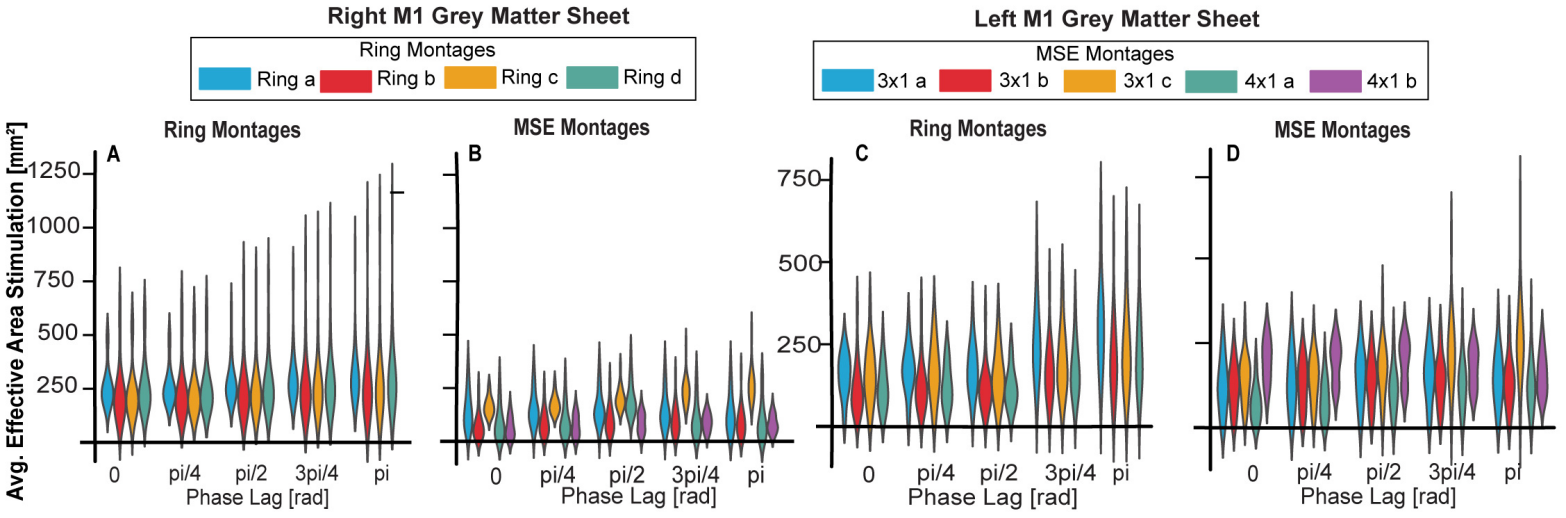
Average area of effective stimulation for the right M1 with ring montages (A) and MSE montages (B), and for the left M1 with ring montages (C) and MSE montages (D).

As the third key question, we investigated the extent of tissue stimulated in the left and right M1s for each montage and how this metric varied across phase lags. Figure 6 illustrates the effective area of stimulation for the right (panels A-B) and left (panels C-D) M1s (for details on each participant’s measures, see Supplemental Material 1, Fig. S6). The average metrics are listed in Table 4. For all montages, the effective area of stimulation significantly varied with the phase lag in the left hemisphere, right hemisphere, and average of both hemispheres (all corrected p-values $<10^{-3}$ , WHR >204729 for the left M1, WHR >99750 for the right M1, and WHR >1540601 for the average of both M1s).

**Table 4.** Overview of population-average effective area of stimulation per montage.

	3 × 1a	3 × 1b	3 × 1c	4 × 1a	4 × 1b	Ring a	Ring b	Ring c	Ring d
Avg. effective area left M1 [${\text{mm}}^{2}$]	136	129	181	104	178	**217**	151	188	152
Diff max. and min. effective area left M1 [${\text{mm}}^{2}$]	319	298	645	347	**225**	620	543	558	518
Avg. effective area right M1 [${\text{mm}}^{2}$]	150	93	208	101	82	**287**	255	260	283
Diff max. and min. effective area right M1 [${\text{mm}}^{2}$]	352	324	411	318	**182**	727	910	938	938

The average/difference between maximum and minimum across all phase lags are shown. The best montage for every measure (largest mean and smallest difference) is shown in bold.

### 3.5 Differences to the optimal E-field directions change with phase lags

The fourth key question examined the direction of E-fields. Figure 5 illustrates the DotP between E-fields with a phase lag of zero and the other phase lags for both the grey matter sheet of the M1s (panels M-N) and the surrounding grey matter sheet (panels O-P) (see Table 5 for average measures, and Supplemental Material 1, Fig. S7 for participant-specific DotP details). The ΔDotP showed a significant deviation from a uniform distribution for all montages (all corrected p-values $<10^{-3}$ , WHR >84.53 for the M1s, and WHR >6.75 for the surrounding regions).

**Table 5.** Overview of population-average ΔDotP.

	3 × 1a	3 × 1b	3 × 1c	4 × 1a	4 × 1b	Ring a	Ring b	Ring c	Ring d
Avg. ΔDotP									
Surrounding regions	0.065	0.058	**0.045**	0.074	0.116	0.064	0.076	0.062	0.081
Diff. ΔDotP max. - min.									
Surrounding regions	0.417	0.324	**0.293**	0.494	0.397	0.363	0.345	0.338	0.362
Avg. ΔDotP M1s	**0.093**	0.147	0.101	0.126	0.107	0.118	0.109	0.119	0.110
Diff. ΔDotP Max. - Min. M1s	**0.177**	0.226	0.189	0.310	0.184	0.376	0.219	0.232	0.223

The average/difference between the maximum and minimum across all phase lags are shown. The best montage for every measure (smallest value) is shown in bold.

### 3.6 Substantial inter-individual variability

As the fifth key question, we quantified the variability of the investigated E-field properties across individuals. Table 6 lists the calculated CVs for all metrics. For all montages, the ΔDotP for the surrounding grey matter sheet exhibited the highest variability between the metrics, with an average CV, across all montages, of 98%. Also in the M1s, the average CV of the ΔDotP was 48%. Additionally, other metrics such as the effective area showed substantial inter-individual variability.

**Table 6.** Overview of the CV for the different E-field characteristics, for each montage and for an average over all the montages.

	3 × 1a	3 × 1b	3 × 1c	4× 1a	4 × 1b	Ring a	Ring b	Ring c	Ring d	Avg. Montages
CV avg. peak magnitude surrounding regions [%]	30	60	**21**	32	24	31	29	29	27	31
CV avg. peak magnitude M1s [%]	29	32	**21**	31	31	30	28	28	27	29
CV avg. peak normal surrounding regions [%]	29	29	**20**	31	23	31	28	29	27	28
CV avg. peak normal M1s [%]	26	32	**20**	27	27	30	27	27	27	27
CV avg. RDM surrounding regions [%]	19	16	**15**	22	26	20	25	25	24	21
CV avg. RDM M1s [%]	20	19	21	22	26	18	**18**	19	19	20
CV avg. ΔDotP surrounding regions [%]	106	92	96	112	**63**	102	106	103	104	98
CV avg.Δ DotP M1s [%]	45	**30**	53	48	45	59	52	49	52	48
CV avg. effective area left M1 [%]	56	52	57	68	**34**	53	65	56	58	55
CV avg. effective area right M1 [%]	58	73	**32**	81	59	40	62	59	55	58

The montage with the lowest CV for each measure is shown in bold.

### 3.7 Potential for individualisation of montages

To evaluate the potential for individualising montages, we focused our analysis on the target area, the grey matter sheet of the M1s. For each metric, we first identified the montage that delivered the best results for the highest number of individuals, providing a group-level overview (see Supplemental Material 2). We then selected individual montages for every participant to assess an improvement in the overall RDM and ΔDotP as well as their variability.

At the group level, we compared all montages, including the MSE and ring montages, to determine which configuration was optimal for the E-field characteristics. The MSE 3 × 1c montage showed the greatest peak magnitude in 66.7% of the individuals. For the peak magnitude normal component, montages 3 × 1c and 4 × 1b showed the highest values for 44.4% of the individuals, each. Regarding the E-field redistribution, there was low variability, with 4 × 1b showing the lowest value for 94.4% of the individuals. Differences in the intended direction were observed across individuals, with the 3 × 1b montage showing the lowest ΔDotP for 44.4% of the individuals. The effective area of stimulation showed high variability in the left M1, with 38.9% of individuals showing the highest value with Ring a. In contrast, 83.3% of the individuals showed the highest value in the right M1 with the same montage.

When considering only MSE montages, the MSE montage 3 × 1c produced the highest peak magnitude in the majority of individuals (66.7%). For the peak magnitude of the normal component of the E-field, montages 3 × 1c and 4 × 1b showed the highest values for 44.4% of individuals, each. The 4 × 1b montage also exhibited the most stable E-field redistribution (lowest RDM) for the largest fraction (94.4%) of the individuals. However, the montage with the lowest ΔDotP varied across individuals, with 3 × 1a showing the highest consistency for 44.4% of the individuals. Notably, montage 3 × 1c yielded the largest effective stimulated area for most individuals, for the left M1 (44.4%) and for the right M1 (72.2%).

For the ring montages, most individuals (94.44% and 88.8%) showed the highest peak and normal component magnitudes for Ring a, respectively. The lowest RDM values were observed for montage Ring c in 44.4% of the individuals. Additionally, the montage with the lowest ΔDotP in most individuals was the Ring d montage (44.4%). For the effective area of stimulation, Ring a showed the highest values in both M1s, with 83.3% of the individuals for the left M1 and 88.9% for the right M1.

To estimate the potential for individualisation of montages, we selected individual montages to minimise the ΔDotP or RDM for each individual and compared the results to the best-performing fixed montage. When optimising the ΔDotP, the average ΔDotP decreased from 0.15 to 0.08, and its CV decreased from 30% to 24%. When optimising the RDM, the average RDM decreased from 0.26 to 0.15, and its CV increased from 18% to 19%.

In summary, we found that certain fixed montages outperformed others, but no single montage was optimal for all metrics or all individuals. By selecting individual montages, the average values of RDM and ΔDotP decreased, indicating that the resulting E-field characteristics became more similar to the ideal case. Additionally, individualisation of montages reduced the variability of the ΔDotP.

## 4 Discussion

We systematically quantified the impact of varying phase lags on the E-field characteristics of ds-tACS, including its magnitude, redistribution, direction, and effective area of stimulation. We observed significant differences in all key characteristics of the E-field across the phase lags and substantial inter-individual variability in all characteristics. When individualising montages to optimise the most critical characteristics, average direction and average redistribution, these metrics could be improved by 40-50%. The analyses presented in this study are not limited to modelling ds-tACS; they can also be applied when stimulating more than two regions simultaneously using HD montages, enabling systematic optimisation of multi-site interventions.

Our findings indicate small but significant differences in the E-field magnitude and its normal component across different phase lags. Qualitatively, these findings have already been described by Saturnino et al. (2017), who showed that for both a 4 × 1 and a ring montage, the E-field magnitude and focality varied between in-phase (zero) and anti-phase (π) conditions. Similarly, Alekseichuk et al. (2019) experimentally confirmed E-field magnitude differences between phase lags zero and π, although their study did not use an HD montage. We extend these findings to phase lags other than zero and π, and quantify the results for the case of targeting the M1s. Choosing arbitrary, for example physiologically plausible phase lags for ds-tACS may potentially boost the effects of ds-tACS (Gann et al., 2025; Schwab et al., 2021). In the future, dynamic models may be used to estimate optimal phase lags for each individual (Schwab, 2025; Schwab et al., 2021).

As noted earlier (Saturnino et al., 2017), wider montages were associated with higher E-field magnitudes but also lower separability of E-fields (larger RDM and ΔDotP). This suggests a general trade-off between E-field magnitude and spatial specificity. Thus, for every experimental study, this trade-off should be considered. For example, clinical studies may profit from relevant effect sizes and may choose for larger E-fields at the cost of overlapping E-fields. In contrast, basic science studies with many participants, which can deal with lower effect sizes, may rather profit from well-separated E-fields, avoiding confounding factors.

Potential differences in E-field magnitude and other characteristics across phase lags are important when interpreting ds-tACS results, as they may confound phase-specific effects on functional connectivity (FC) by modulating other neural signals such as EEG power or BOLD activity. Indeed, several studies have reported phase-dependent modulation of both FC and these neural signals (Preisig et al., 2021; Violante et al., 2017; Zhang et al., 2023). However, the relatively stable E-field magnitudes observed here align with findings from most HD ds-tACS studies that report FC changes without accompanying power modulation (Gann et al., 2025; Reinhart, 2017; Reinhart & Nguyen, 2019; Schwab et al., 2019). Thus, we assume that smaller changes in E-field magnitudes, as observed for HD ds-tACS, have at most minor effects on power modulation, in line with the concept of “effective doses” for tACS (Alekseichuk et al., 2022).

While the functional consequences of these E-field characteristics were not directly assessed in our study, the phase lag–dependent changes in direction are likely to influence FC outcomes. Deviations from the intended E-field direction (ΔDotP) may alter entrainment properties of targeted neural populations. In contrast, it is more challenging to interpret the RDM values, indicating differences in spatial field distributions, and implying that different regions may be stimulated across phase lags. Although the RDM values obtained here suggest only modest spatial deviations, even small differences could introduce considerable variability in stimulation effects. It remains to be shown for every experimental study whether differences in E-field characteristics influence the physiological or behavioural outcomes.

Next to changes across phase lags, also inter-individual variability is relevant for ds-tACS. While certain E-field properties, such as the RDM, were relatively stable across individuals, the ΔDotP and effective area of stimulation showed considerable inter-individual variability, next to the changes across phase lags. Thus, applying the same stimulation settings across individuals may result in inconsistent E-field directions and stimulated areas. In particular, the E-field direction is crucial for connectivity modulation, and unintended deviations from the intended direction could impede effective FC modulation. Therefore, individualised optimisation targeting ΔDotP could substantially improve the consistency and efficacy of ds-tACS across participants. In contrast, while the impact of the RDM on FC is less clear, the relatively low inter-individual variability in the RDM suggests that individualisation based on this metric alone may be less critical.

We, therefore, tested if individualisation of montages can also be done for E-field properties that are specific to ds-tACS, the redistribution of E-fields across phase lags, and the direction of E-fields. Several studies already have demonstrated that differences in individual anatomy can lead to substantial variability in the magnitudes and focality of tACS, and that those features can be optimised using established pipelines (Kasten et al., 2019; Laakso et al., 2015; Opitz et al., 2016; Preisig et al., 2021; Tseng et al., 2018). In contrast, our approach for optimising the RDM and ΔDotP is based on the pre-selection of a number of montages which are all explicitly simulated, allowing to optimise for any arbitrary feature. We showed that individualising for the optimal direction—a critical characteristic of ds-tACS, which is intended to drive the changes in FC—clearly improves the fit to the intended direction, and reduces inter-individual variability. Such an individualisation may therefore enhance the precision and efficacy of ds-tACS.

Our study also shows some limitations. First, although our sample of 18 individuals was evenly distributed across age groups and balanced for biological sex, it may not fully capture the anatomical variability present in the general population. Similarly, our analysis was limited to healthy young individuals, and caution is warranted when generalising the findings to clinical or aging populations, where brain pathology may alter current flow. Nevertheless, we provide a methodology that can be used on any individual cohort and suggest a way to individualise montages. This is possible via the simulation of a range of montages, where the optimal montage can be chosen for each individual.

Second, although our SimNIBS head models are detailed, they cannot capture dynamic effects, such as changes in skin conductivity due to sweating or movement of the brain within the CSF. Third, our optimisation approach, which is based on the explicit simulation of a number of pre-selected montages per individual, is computationally expensive. However, in the future, machine learning may help estimate individual E-fields without explicitly simulating them, strongly reducing the computational burden. Finally, our study described E-field characteristics, but cannot directly draw conclusions on the functional implications of these E-fields. Future studies may experimentally investigate whether the observed phase-lag-dependent differences in E-field characteristics translate to measurable differences in physiological or behavioural outcomes.

## 5 Conclusion

Our findings revealed that variations in the phase lag of HD ds-tACS significantly impact key E-field properties, introducing potential confounding factors for experimental designs. Most prominently, the relative direction—the main factor intended to drive changes in FC—was different from the intended relative direction and showed considerable inter-individual variability. The observed inconsistent E-field properties complicate the interpretation of physiological changes under ds-tACS.

In summary, we recommend considering phase-dependent changes in E-field characteristics like magnitude, distribution, direction, and area of stimulation when designing ds-tACS studies, as they may potentially lead to unintended effects on neural activity. Using our methods, these properties can be simulated for any arbitrary phase lag, and can be statistically related to experimental outcomes. We furthermore propose that montages can be individualised based on a pre-selection of possible montages to optimise E-field characteristics for ds-tACS, and in particular, to achieve the desired direction of E-fields.

## Supplementary Material

Supplementary Material 1

Supplementary Material 2

## Data Availability

The code is available at https://gitlab.utwente.nl/bss_development/neuro/acs/EfieldPropertiesDualtACS. The raw data are available in the HCP database (Van Essen et al., 2013) under the S1200 database.

## Appendices

### A. Montages and Electrodes Characteristics

**Table A7.** Sizes of the ring montages used in the study.

Ring montages	Radius central electrode (mm)	Radii surrounding electrode (mm)
Ring a	12.5	Inner: 46, Outer: 57.5
Ring b	10	Inner: 37.5, Outer: 50
Ring c	12.5	Inner: 39, Outer: 50
Ring d	17	Inner: 37.5, Outer: 50

The table provides the radius of the central electrode and the inner and outer radii of the surrounding electrodes for different ring montage configurations.

**Table A8.** Positions of electrodes for the MSE montages.

MSE montages	Position central electrodes	Position surrounding electrodes
3in1a	C3, C4	LH: C1, FC3, CP3. RH: C2, FC4, CP4
3in1b	CCP3h, CCP4h	LH: CCP1h, FCC3h, CPP3h. RH: CCP2h, FCC4h, CPP4h
3in1c	C3,C4	LH:F3, T7, P3. RH: F4, T8, P4
4in1a	C3, C4	LH: C1, FC3, C5, CP3. RH: C2, FC4, C6, CP4
4in1b	C6, C5	LH: FT7, FC3, CP3,TP7. RH: FT8, FC4, CP4,TP8

The table lists the central and surrounding electrode positions for different montage configurations, using the SPM12 EEG system.

**Table A9.** Material and electrical characteristics of the electrodes and gel layers used in the study.

Parameter	Values
Thickness electrodes	1 mm
Thickness gel layer	2 mm
Conductivity gel layer	1 S/m
Conductivity ring electrodes and 3 × 1b (silicone rubber)	29.4 S/m
Conductivity MSE (Ag/AgCl) except 3 × 1b	13.6 S/m

The table lists the material composition, thickness, and conductivity values of both the MSE (Ag/AgCl) and the ring electrodes (rubber). The gel layer properties, including thickness and conductivity, are also provided.

### B. Analysed Regions

Table A10 presents the 14 unilateral regions used for the analysis, all of which were derived from the Glasser Atlas (Glasser et al., 2016). The analysis included both hemispheres, resulting in 28 brain regions.

**Table A10.** Regions of the “surrounding grey matter sheet”.

Name regions	Area description
1	Area 1
2	Area 2
3a	Area 3a
3b	Primary sensory cortex
55b	Area 55b
6a	Area 6 anterior
6d	Dorsal area 6
6r	Rostral area 6
6v	Ventral area 6
FEF	Frontal eye fields
OP1	Area OP1/SII
PF	Area PF complex
PFop	Area PF opercular
Pft	Area Pft

All regions were included bilaterally.
