# Supplementary material for: Variable E-field properties of dual-site tACS with phase lags
Source: Imaging Neurosci (Camb). 2026 Jan 5;4:IMAG.a.1068. doi: 10.1162/IMAG.a.1068 (PMC12770909; doi:10.1162/IMAG.a.1068)
Supplement: Supplementary Material 1 [file IMAG.a.1068_supp1.pdf]

Variable E-field properties of dual-site tACS with phase lags

Silvana Huertas-Penen, Maria Carla Piastra, Oula Puonti, Bettina C. Schwab*

*Corresponding author

Supplemental Material 1

A. IDs of HCP datasets

Table S1: IDs of the datasets used for this study. The data was obtained from the HCP (Van Essen et al. 2013).

IDs	Age Group	Biological Sex
147636	22-25	F
350330	22-25	F
970764	22-25	F
558657	22-25	M
728454	22-25	M
886674	22-25	M
153126	26-30	F
168947	26-30	F
257946	26-30	F
138332	26-30	M
144933	26-30	M
329844	26-30	M
146735	31-35	F
360030	31-35	F
481042	31-35	F
152225	31-35	M
723141	31-35	M
969476	31-35	M

B. Calculation of the Ideal DotP values

The ideal DotP is determined under the assumption that two distinct areas are stimulated without leakage or overlap of the E-fields between the electrodes. Under these conditions, the E-fields in both areas were considered to be independent.

For ds-tACS, a phase shift is applied to the current in one area ($Area_a$), whereas no phase shift was applied to the current in the other area ($Area_b$). The applied currents are given by

$$I_a(t) = \sin(2\pi f t),$$

$$I_b(t) = \sin(2\pi f t + \phi),$$

When calculating the DotP, we compared the direction of the normal component of the E-field at a given phase lag with that of the E-field at zero phase lag. In an ideal case, the direction of the normal E-field component is directly related to the applied current. Specifically, a positive current generates a positive normal E-field component (oriented inward towards the grey matter), whereas a negative current produces a negative

normal E-field component (oriented outwards from the grey matter) (Antonenko et al., 2019).

In all cases, $Area_a$ did not receive a phase shift. Therefore, the normal E-field component in this area remained unchanged between the zero-phase and phase-lagged models. Consequently, the DotP for this area is always 1. In contrast, in $Area_b$, where a phase shift is applied, the direction of the normal E-field component varies over time as a function of the phase shift ϕ:

$$IDotP_{0,\phi} = \frac{1}{2}\left(\frac{1}{T}\int_0^T sign[sin(2\pi ft)] \cdot sign[\sin(2\pi ft + \phi)]\, dt + \frac{1}{T}\int_0^T sign[sin(2\pi ft)] \cdot sign[\sin(2\pi ft)]\, dt\right)$$

$$= \frac{1}{2}\left(\frac{1}{T}\int_0^T sign[sin(2\pi ft)] \cdot sign[\sin(2\pi ft + \phi)]\, dt + 1\right)$$

Here, T denotes the period of the sinusoidal function, and the division by two accounts for averaging across the two hemispheres or brain areas analysed. Importantly, the phase shift ϕ is applied only in one area.

To illustrate this, we calculated the ideal DotP for the π/2 phase lag condition. In $Area_a$ the current remained identical in both the zero-and π/2-phase lag models, resulting in a constant direction of the normal E-field component throughout the sinusoidal cycle (Figure S1.A). Therefore, the DotP for this region is 1 ($IDotP_a$). In contrast, in $Area_b$, the sinusoidal currents for the zero-phase lag $\sin(2\pi ft)$ and $\sin(2\pi ft + \pi/2)$ differ in phase. Owing to this phase shift, the direction of the normal E-field component in $Area_b$ changes over time. In the ideal case, at certain time points, both currents share the same sign (either positive or negative; see Figure S1.B), implying that the normal E-field components are aligned, yielding an ideal DotP value of +1. At other times, the currents have opposite signs (one positive and one negative), indicating anti-alignment and resulting in an ideal DotP value of -1. At four specific time points, the current was zero, resulting in an ideal DotP value of 0.

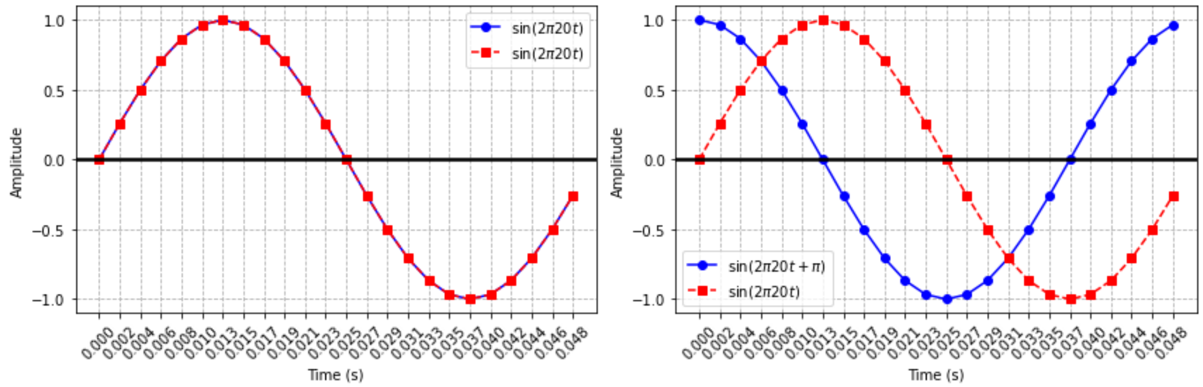

Figure S1: Example for computing the DotP under ideal conditions and a phase lag of $\pi/2$. A: Currents for $Area_a$. B: Currents for area $Area_b$.

In our simulations, we discretised the sinusoidal wave into 24 time steps. The ideal DotP was computed as follows:

$$IDotP = \frac{\sum_{i=1}^{n}(sign[I_a(t_i)] \cdot sign[I_b(t_i)])}{n}$$

where:

- $I_a(t_i)$ and $I_b(t_i)$ represent the current at time step t_i for the two regions,
- $sign(x)$ denotes the sign of x i.e., $sign(x)$ = +1 if $x > 0$, $sign(x)$ = -1 if $x < 0$, and $sign(x)$ = 0 if $x = 0$,
- n is the total number of time steps.

For the discretised sinusoidal example, the IDotP in $Area_b$ becomes

$$IDotP_b = \frac{(5\,X + 1) + (5\,X - 1) + (5\,X + 1) + (5\,X - 1) + (4\,X\,0)}{24} = 0$$

To obtain the ideal DotP, we averaged the ideal DotP values from both areas as follows:

$$\frac{IDotP_a + IDotP_b}{2} = \frac{1 + 0}{2} = 0.5$$

where $IDotP_a$ is the ideal DotP in one area and $IDotP_b$ is the ideal DotP in the other area. Thus, the ideal DotP for the π/2 phase lag condition is 0.5 (See Table S2 for the ideal DotP of all the analysed phase lags).

Table S2: Ideal DotP values for each phase lag.

Phase lags (radians)	Ideal DotP
π/4	0.75
π/2	0.5
3π/4	0.25

C. Spherical head model

The spherical head model had a radius of 95 mm. Two montages were positioned at the maximum distance from each other on the sphere, each including a central circular electrode with a radius of 5 mm and a surrounding ring-shaped electrode with outer and inner radii of 15 and 10 mm, respectively. These montages were very small compared to the size of the sphere (Figure S2.A). The results of the spherical head models are shown in Figure S2.B-D.

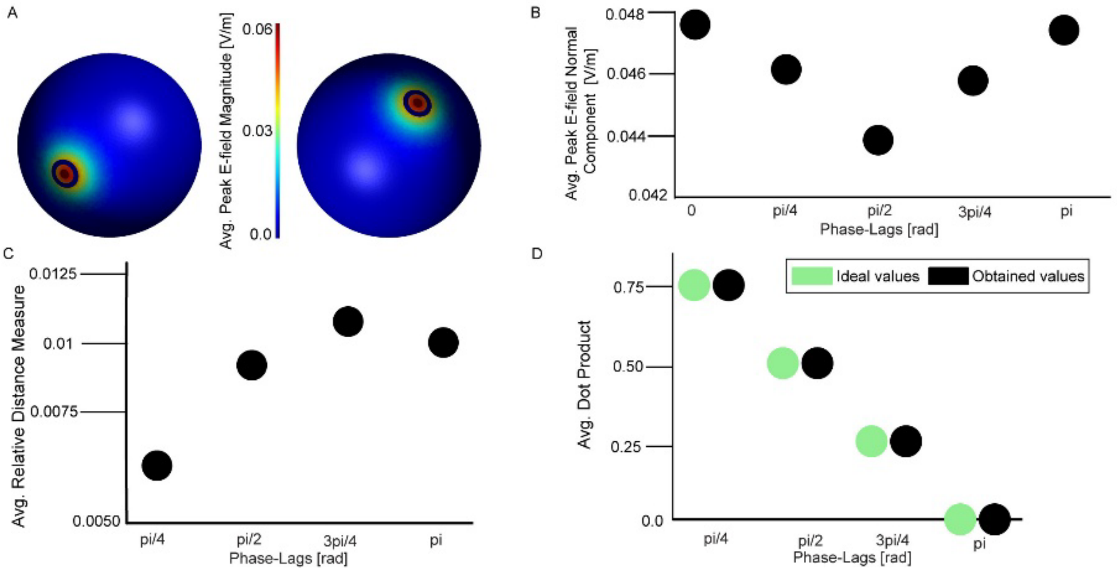

Figure S2: Near-ideal values for a sphere. A) Small ring electrodes were used on a large sphere, leading to low average E-field magnitudes. B) The average E-field magnitude normal component barely varies across the phase lags. C) The average RDM was low across all phase lags. D) The DotP (black) is close to the ideal (green) values for all phase lags.

D. Detail results for all metrics

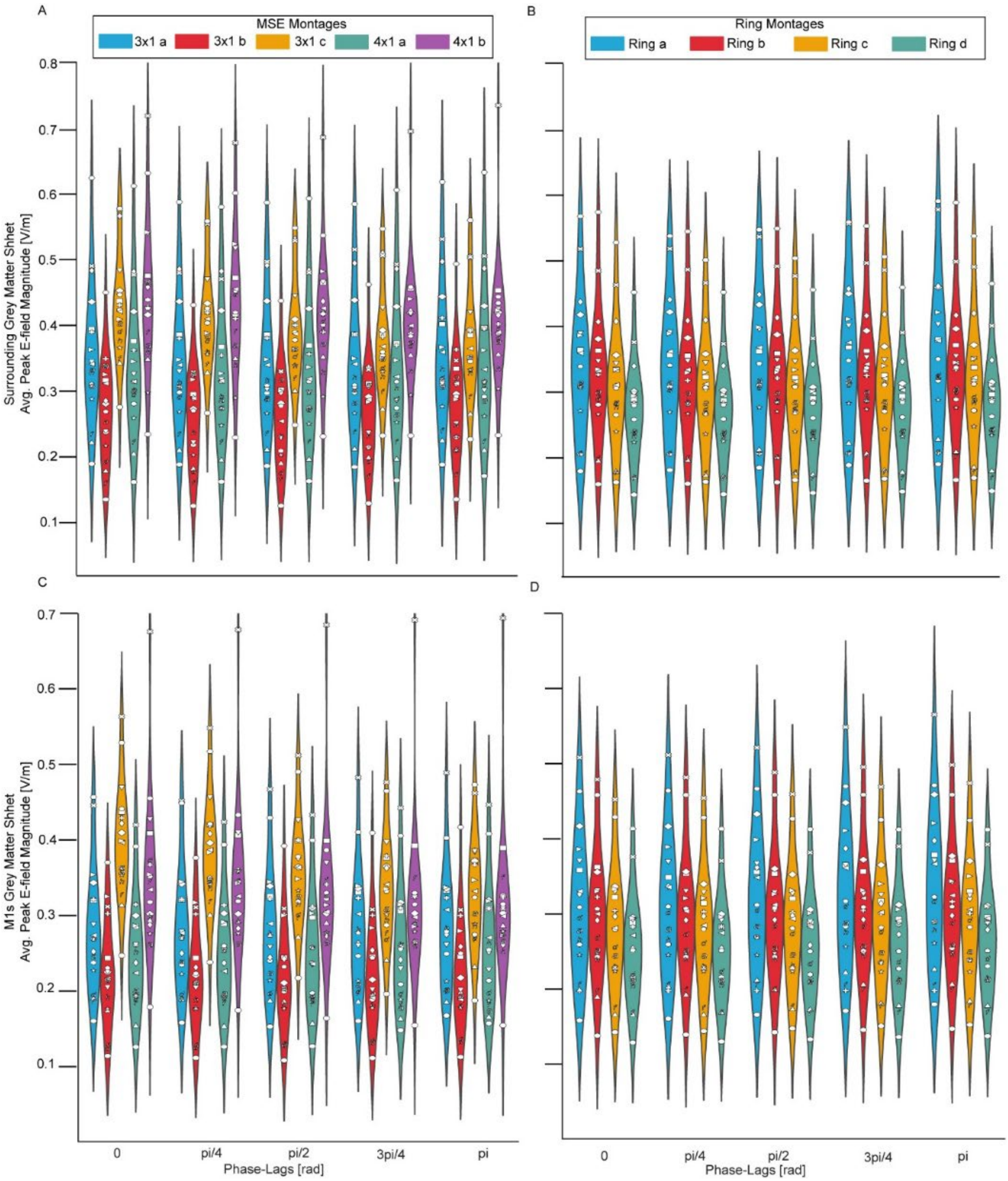

Figure S3: Maximum peak E-field magnitude for the *MSE* montages (A, C) and *ring* montages (B, D) in the surrounding grey matter sheet (A, B) and the M1s (C, D) . The data from each individual are depicted as unique symbols.

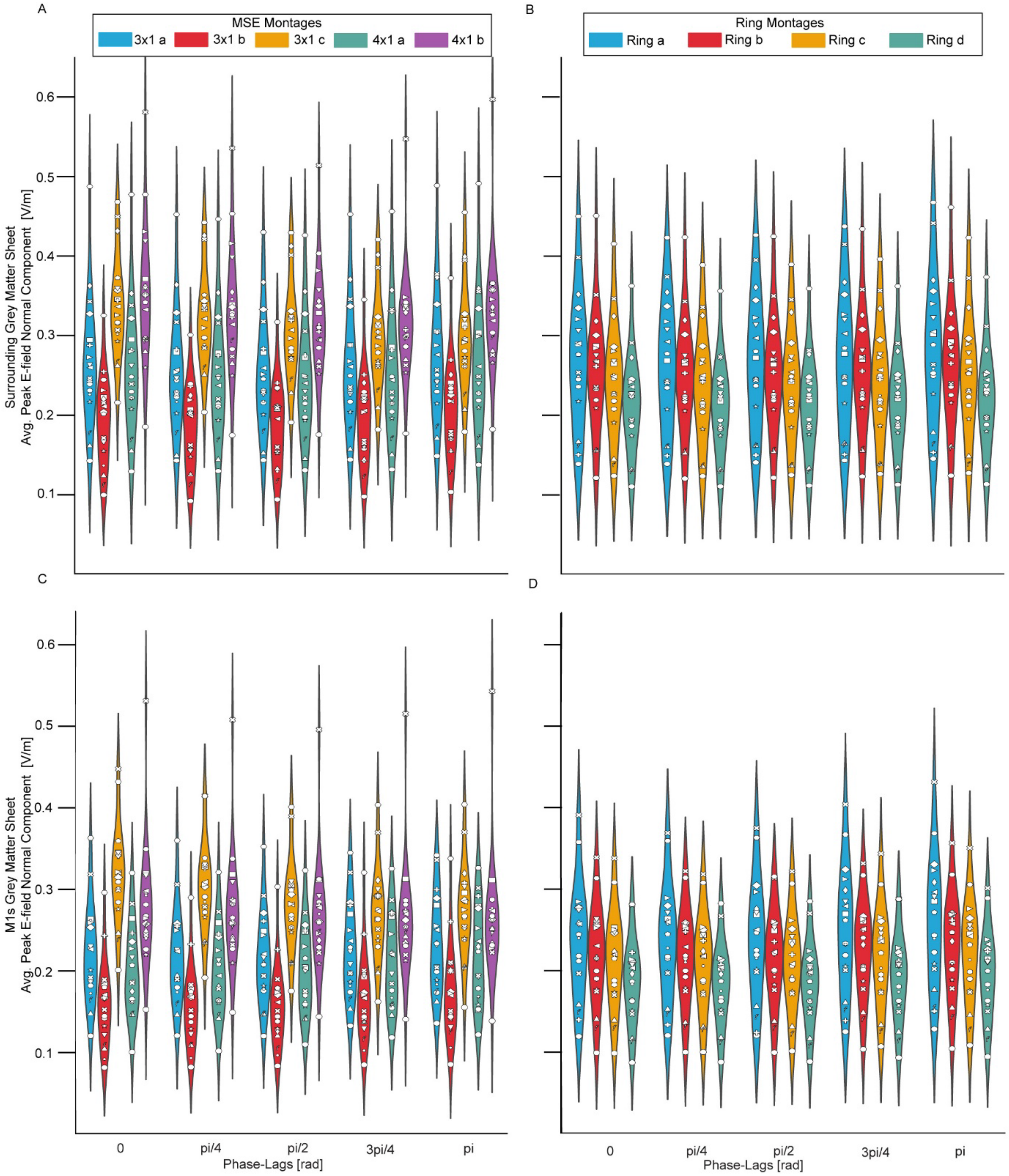

Figure S4: Maximum peak E-field normal components for the *MSE* montages (A, C) and *ring* montages (B, D) in the surrounding grey matter sheet (A, B) and the M1s (C, D). Data from every individual is depicted as a unique symbol.

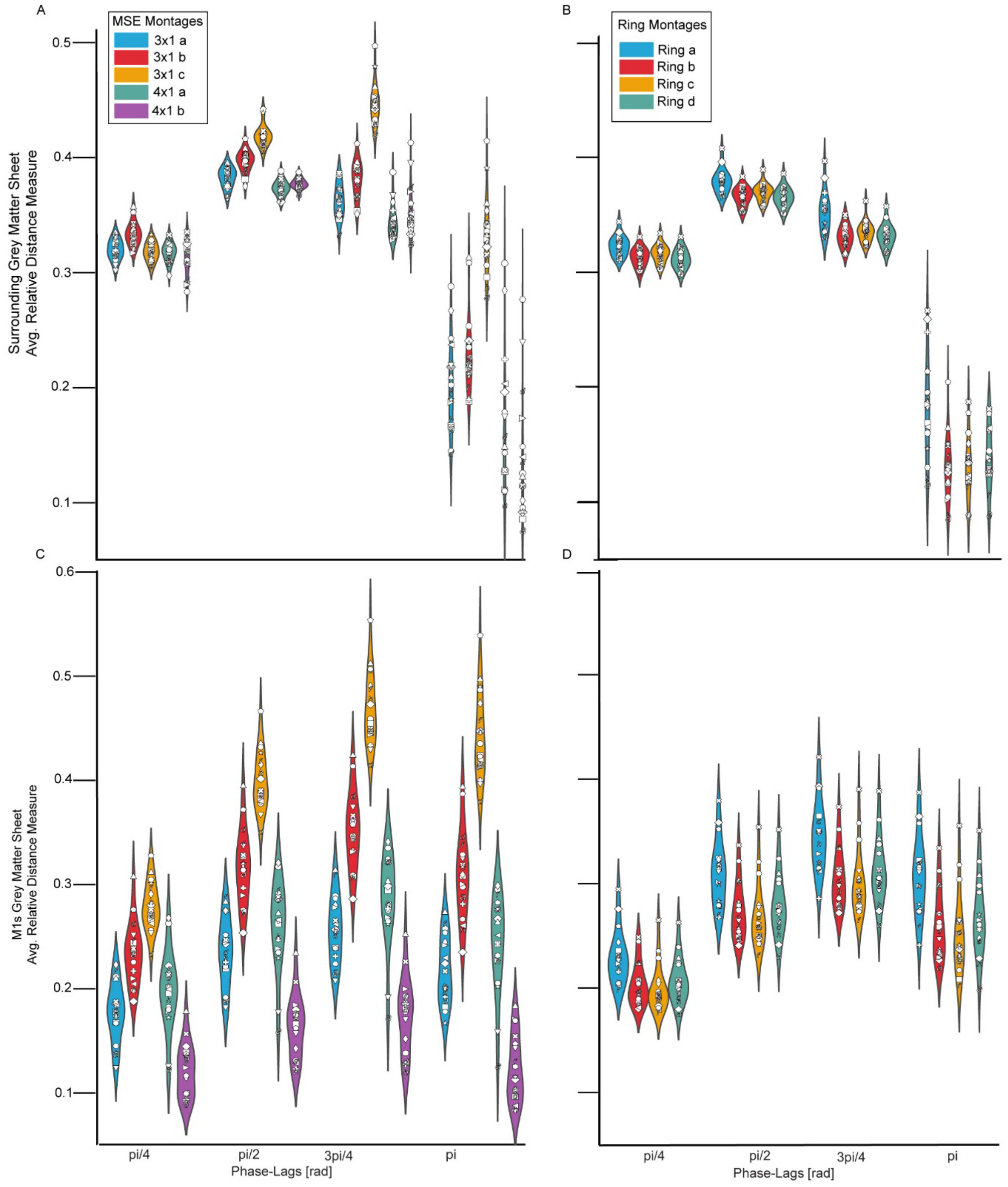

Figure S5: Average RDM for the the *MSE* montages (A, C) and *ring* montages (B, D) in the surrounding grey matter sheet (A, B) and in the M1s (C, D). The data from each individual are depicted as unique symbols.

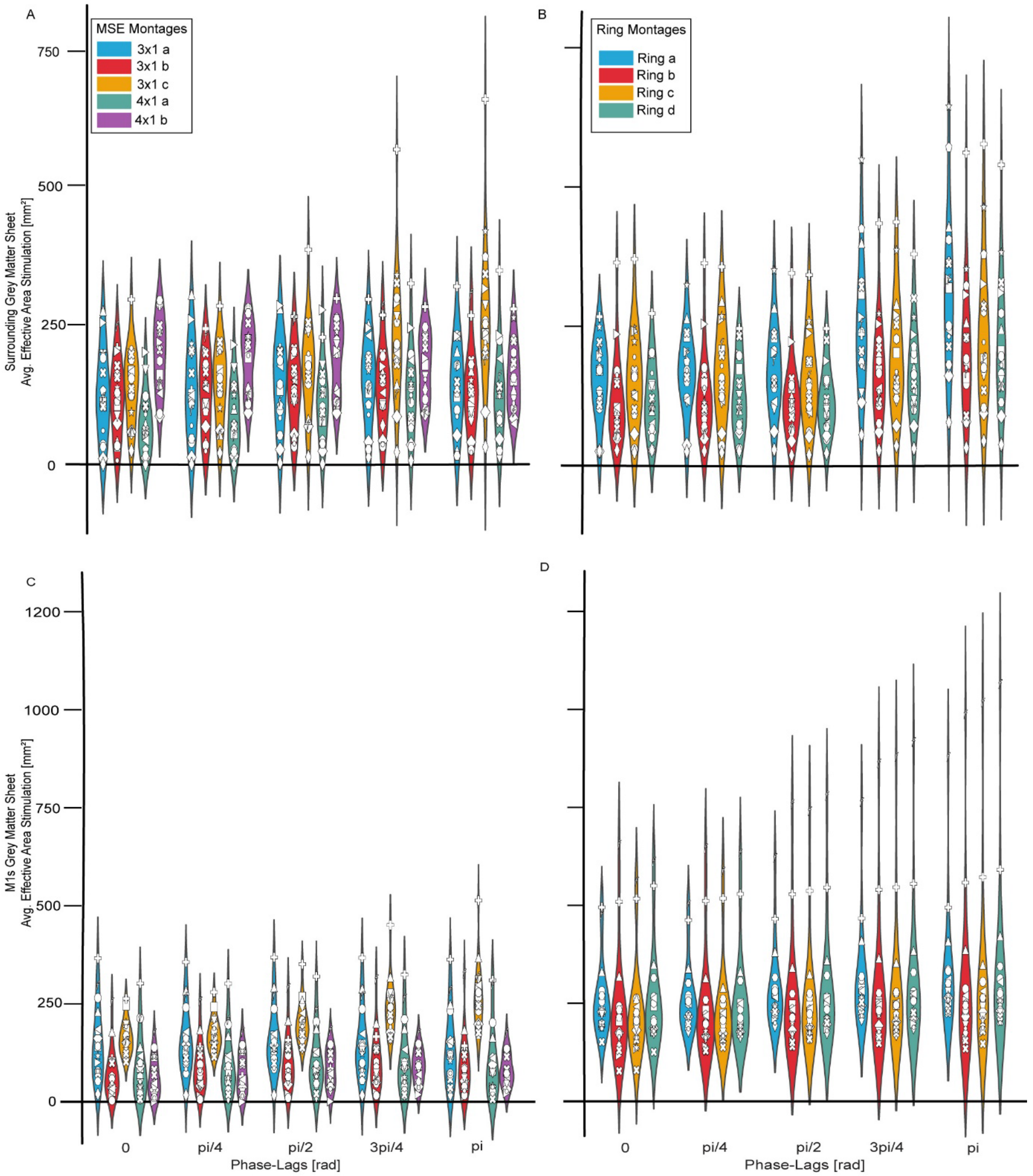

Figure S6: Average area of effective stimulation for the *MSE* montages in the left M1 (A) and right M1 (C), and for the *ring* montages in the left M1 (B) and right M1 (D). Data from every individual is depicted as a unique symbol.

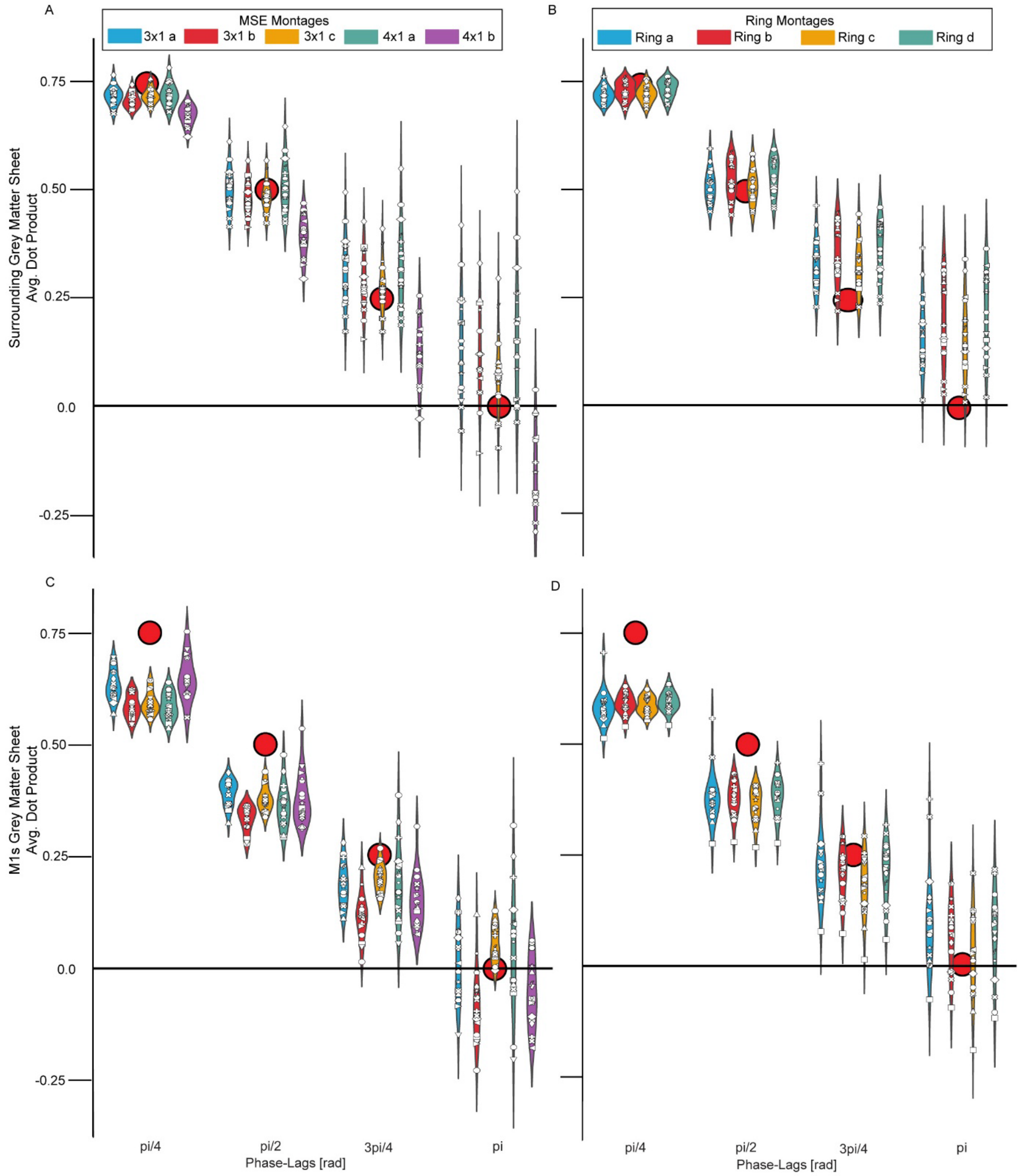

Figure S7: Average DotP for the *MSE* montages (A, C) and *ring* montages (B, D) in the surrounding grey matter sheet (A, B),and in the M1s (C, D). Data from every individual is depicted as a unique symbol. The red dots represent the ideal DotP values.

References Supplementary material:

Antonenko, D., Thielscher, A., Saturnino, G. B., Aydin, S., Ittermann, B., Grittner, U., & Flöel, A. (2019). Towards precise brain stimulation: Is electric field simulation related to neuromodulation? Brain stimulation, 12 (5), 1159–1168

Van Essen, D. C., Smith, S. M., Barch, D. M., Behrens, T. E., Yacoub, E., Ugurbil, K., Consortium, W.-M. H., et al. (2013). The Wu-minn human connectome project: An overview. Neuroimage,80, 62–79.
